# Supplementary material for: Clinically relevant combined effect of polygenic background, rare pathogenic germline variants, and family history on colorectal cancer incidence
Source: BMC Med Genomics. 2023 Mar 5;16:42. doi: 10.1186/s12920-023-01469-z (PMC9987090; doi:10.1186/s12920-023-01469-z)
Supplement: Supplementary file 2 — Additional file 2. Supplemental Figures 1–9 and Supplemental Tables 1–7. [file 12920_2023_1469_MOESM2_ESM.pdf]

Clinically relevant combined effect of polygenic background, rare pathogenic germline variants, and family history on colorectal cancer incidence

Supplemental Figures

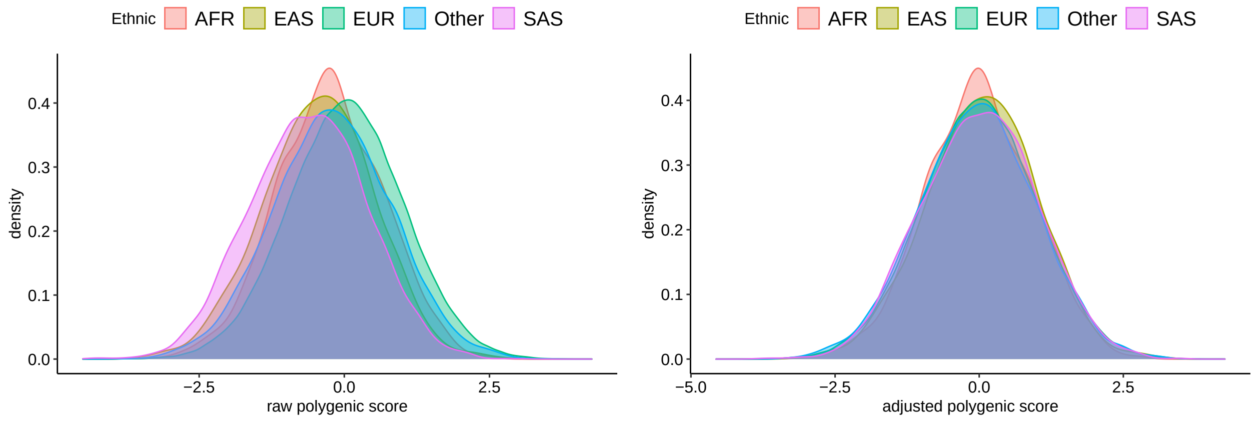

(a) raw PRS **(b)** adjusted PRS

Figure S1: Distributions of the colorectal cancer (CRC) PRS across the estimated genetic ethnicities. Estimated genetic ethnicities were estimated by projecting the samples in the 1000 genome project (1KGP) principal component space while considering the five 1KGP superpopulations as reference (https://github.com/privefl/paper-ancestry-matching/tree/master/code). Distributions of: a) raw PRS; b) adjusted PRS.

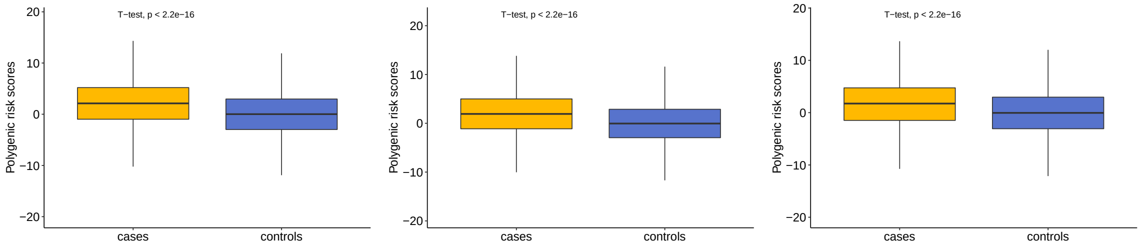

(a) 95 PRS **(b)** 140 PRS **(c)** 50 PRS

Figure S2: PRS among CRC cases versus controls. a) 95 PRS: using the 95 SNPs (Huyghe et al.); b) 140 PRS: using the 140 SNPs (Huyghe et al.); c) 50 PRS: using the 50 SNPs (Briggs et al.).

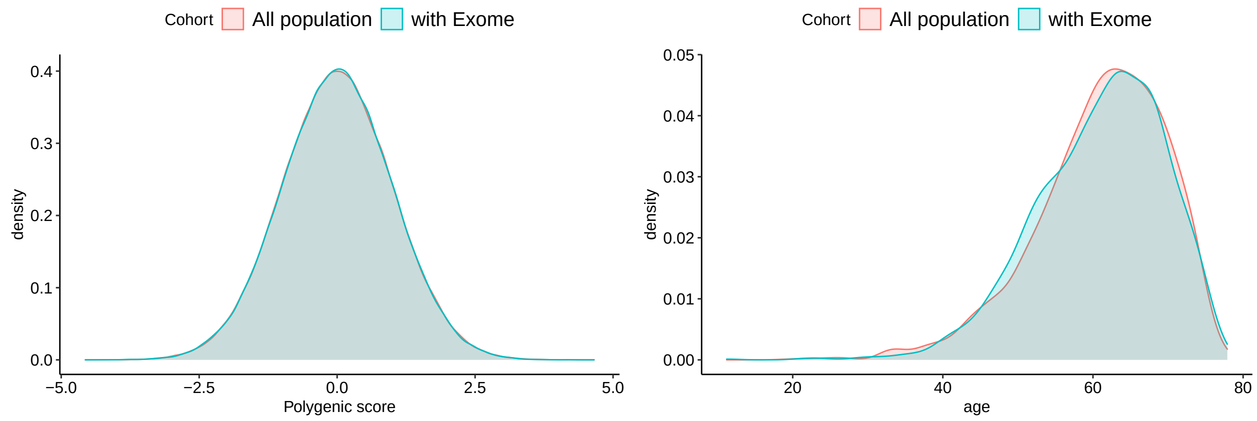

(a) PRS

(b) Age

Figure S3: Distribution of PRS and age across the whole UKBB cohort and the subpopulation with WES data: a) PRS ; b) Age.

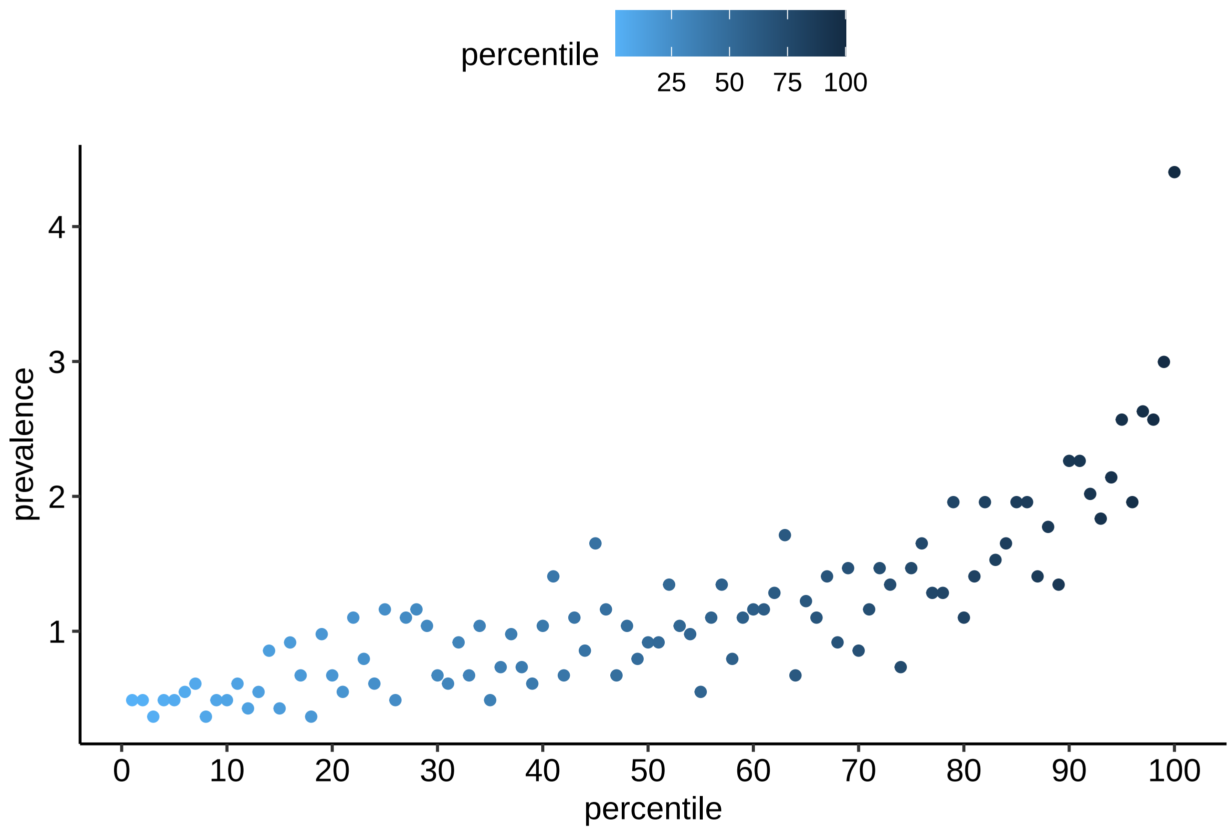

Figure S4: Prevalence of the colorectal cancer (CRC) according to polygenic risk score (PRS) perecentiles.

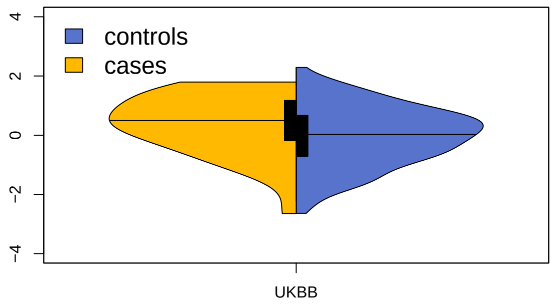

(a) Density plot

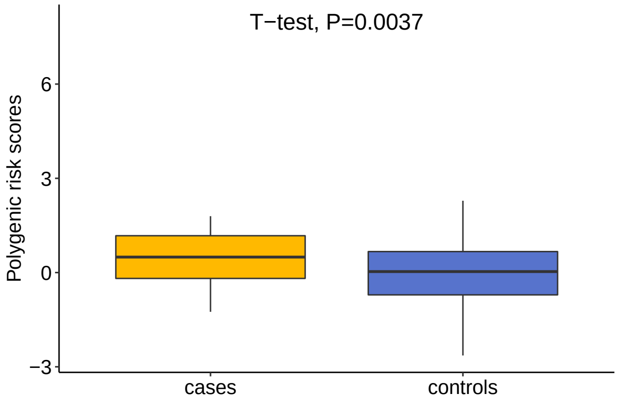

(b) Box plot

Figure S5: PRS among CRC affected (cases) and unaffected (controls) carriers. Density (a) and box plots (b) show the PRS distribution among affected and unaffected carriers. Horizontal line indicates PRS mean in plot (a).

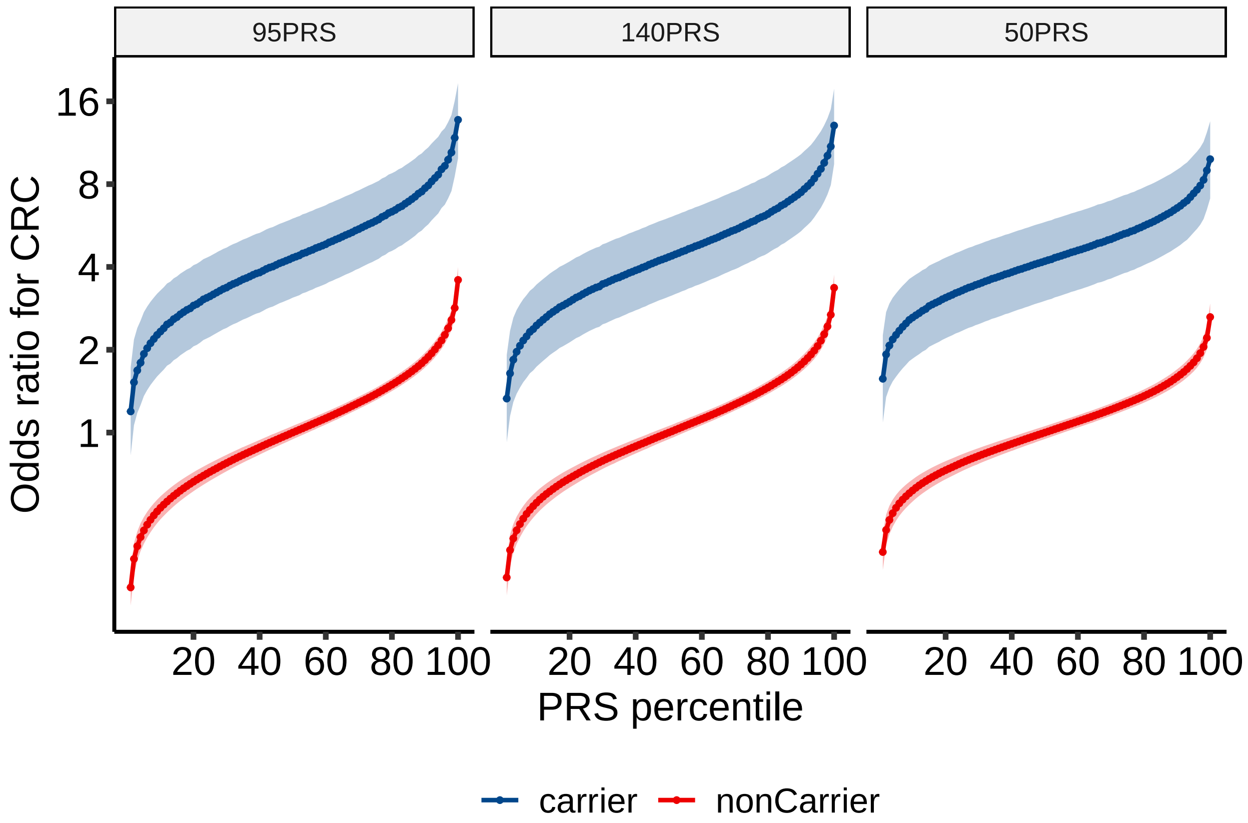

Figure S6: Interplay of heterozygous pathogenic variant (PV) carrier status, and polygenic risk score (PRS) using the three PRS modes. a) 95 PRS: using the 95 SNPs (Huyghe et al.); b) 140 PRS: using the 140 SNPs (Huyghe et al.); c) 50 PRS: using the 50 SNPs (Briggs et al.)

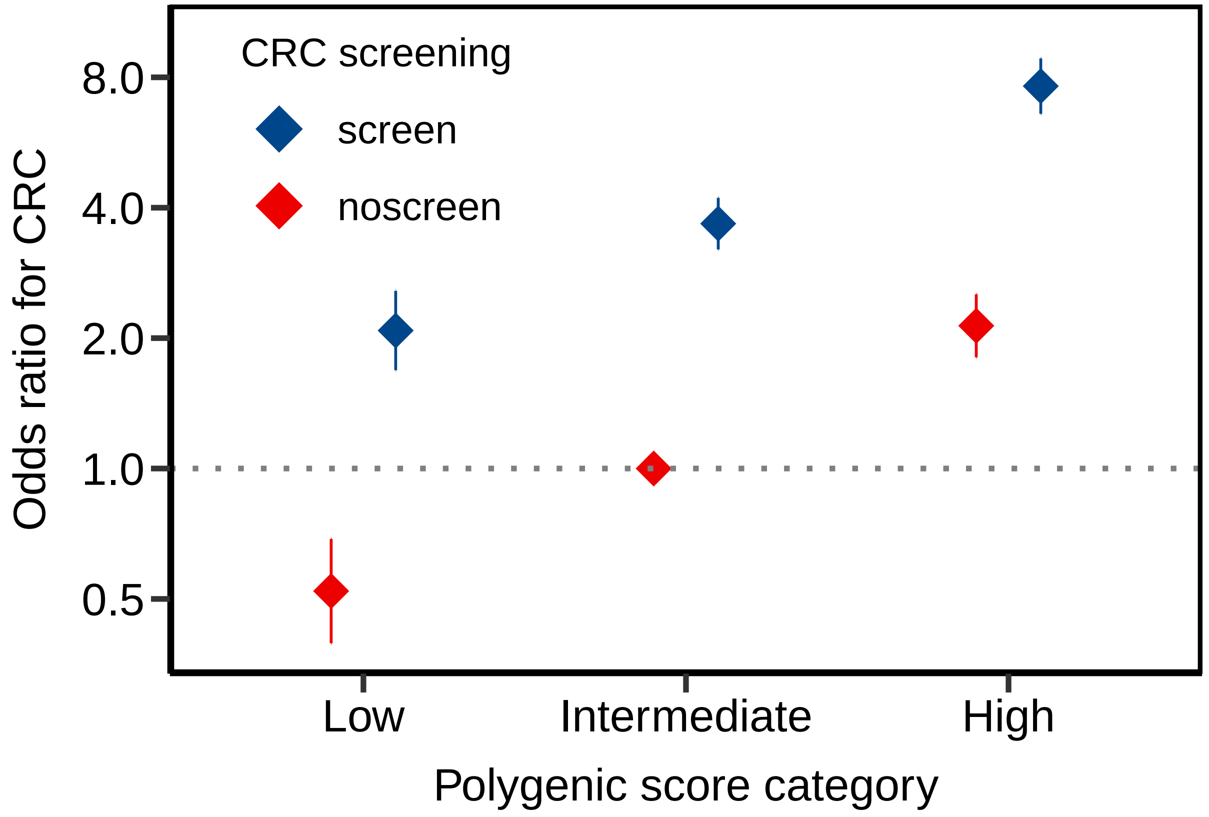

Figure S7: Colorectal cancer (CRC) odds ratio among individuals stratified for CRC screening status and PRS.

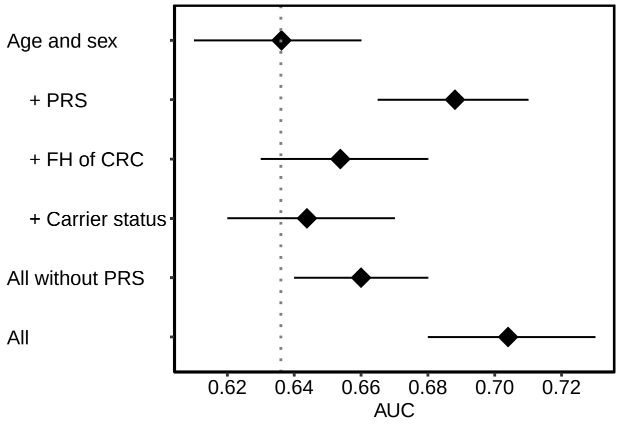

(a) including age and sex in models

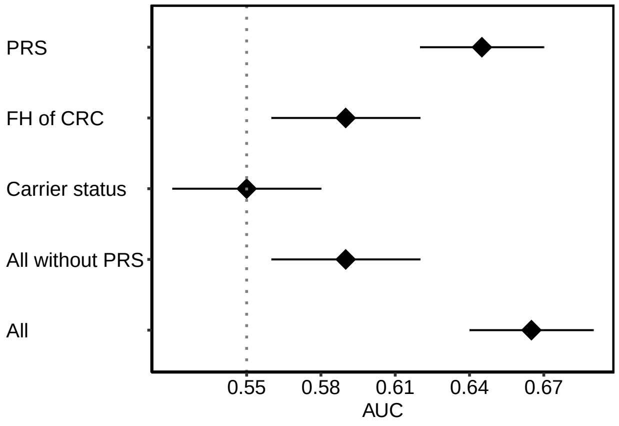

(b) Not including age and sex in models

Figure S8: Model discrimination assessed for combinations of polygenic risk score (PRS), family history of CRC (FH) and carrier. (a) including age and sex in models, (b) Not including age and sex in models

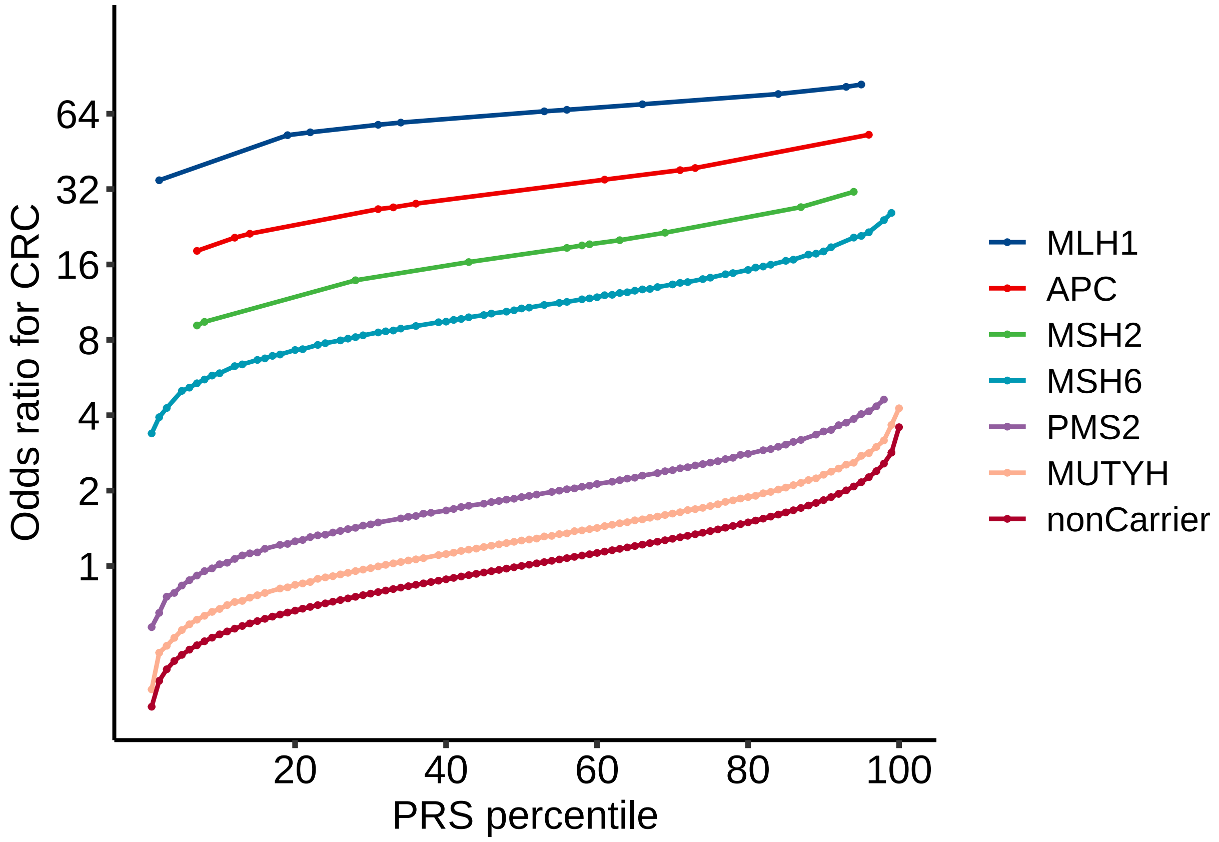

Figure S9: Interplay of heterozygous pathogenic variant (PV) carrier status, family history (FH), and polygenic risk score (PRS) in single genes.

Supplemental Tables

Table S1: Provided as excel file.

Table S2: Number of PV carriers separated by gene and clinical status.

gene	Cases	Controls	Total
APC	3	8	11
MLH1	6	5	11
MSH2	2	11	13
MSH6	14	121	135
PMS2	5	224	229
**Total**	**30**	**369**	**399**
MUTYH*	5	365	370

Note:
* MUTYH variants were included only in the single gene analysis to compare the effect size with the other genes.

Table S3: Interplay of PV, and PRS in CRC. All cases (prevalent and incident cases) were included

	Carrier				nonCarrier			
prs	CI	HR	OR	p.value	CI	HR	OR	p.value
High	74 (70.3-77.7)	11.5 (6.3-20.8)	17.5 (9.0-32.4)	1.6e-17	22 (21.5-22.5)	2.08 (1.9-2.3)	2.08 (1.9-2.3)	7.5e-49
Intermediate	51 (48.5-53.6)	6 (3.7-9.8)	7 (4.2-11.7)	1.1e-13	11 (10.8-11.3)	1	1	
Low	40 (38.0-42.1)	4.3 (1.4-13.2)	3.9 (1.2-12.3)	2.2e-02	6 (5.9-6.1)	0.55 (0.47-0.65)	0.54 (0.46-0.64)	3.6e-14

Note:
CI = Cumulative incidence; HR = Hazard rario; OR = Odds ratio.

Table S4: Interplay of PV, and PRS in CRC. Only incident cases were included

	Carrier		nonCarrier	
prs	OR	p.value	OR	p.value
High	7.0 (2.04-23.7)	1.9e-4	2.11 (1.9-2.4)	3.5e-28
Intermediate	5.6 (2.6-12.3)	1.6e-5	1	
Low	3.5 (1.2-10.4)	9.4e-02	0.49 (0.39-0.61)	4.9e-10

Note:
CI = Cumulative incidence; HR = Hazard rario; OR = Odds ratio.

Table S5: Interplay of PV, and FH in CRC.

	History				nonHistory			
prs	CI	HR	OR	p.value	CI	HR	OR	p.value
High	26 (24.7-27.3)	2.8 (2.3-3.3)	3.1 (2.6-3.8)	1.4e-33	21 (19.9-22.1)	2.1 (1.9-2.4)	2.1(1.9-2.4)	3.1e-44
Intermediate	16 (15.2-16.8)	1.7 (1.4-1.9)	1.9 (1.6-2.18)	2.1e-15	11 (10.5-11.6)	1	1	
Low	8 (7.6-8.4)	0.7 (0.5-1.1)	0.9 (0.58-1.25)	4.2e-01	6 (5.7-6.3)	0.55 (0.49-0.69)	0.6 (0.47-0.67)	1.1e-10

Note:
CI = Cumulative incidence; HR = Hazard rario; OR = Odds ratio.

Table S6: Interplay of PV, FH, and PRS in CRC.

		Carrier				nonCarrier			
prs	history	CI	HR	OR	p.value	CI	HR	OR	p.value
High	History	97.8 (96.0-99.6)	36.3 (13.6-97.1)	39.9 (12.69-125.41)	3e-10	25.5 (24.3-26.8)	2.7 (2.24-3.25)	3.08 (2.55-3.71)	<2e-16
High	nonHistory	62.1 (59.1-65.3)	8.97 (4.25-18.9)	14.56 (6.48-32.73)	9e-11	21 (19.9-22.1)	2.16 (1.94-2.41)	2.16 (1.94-2.41)	<2e-16
Intermediate	History	66.4 (63.2-69.8)	10.1 (4.80-21.3)	15.63 (6.95-35.17)	3e-11	16.3 (15.5-17.1)	1.63 (1.39-1.90)	1.83 (1.56-2.14)	4e-14
Intermediate	nonHistory	42.5 (40.4-44.7)	5.08 (2.63-9.80)	5.56 (2.82-10.94)	7e-07	10.4 (9.9-10.9)	1	1	
Low	History	50 (47.6-52.6)	6.40 (0.901-45.5)	5.78 (0.76-43.95)	9e-02	7.6 (7.2-7.9)	0.73 (0.49-1.08)	0.83 (0.56-1.24)	0.4
Low	nonHistory	35.5 (33.8-37.3)	4.03 (1.01-16.1)	3.74 (0.91-15.43)	7e-02	6.2 (5.9-6.5)	0.58 (0.49-0.69)	0.57 (0.48-0.68)	1e-10

Note:
CI = Cumulative incidence; HR = Hazard rario; OR = Odds ratio.

Table S7: Interplay of PV, PRS and FH in single genes.

gene	history	Low	Intermediate	High
nonCarrier	nonHistory	0.51 (0.46-0.57)	1 (1-1)	1.93 (1.8-2.07)
	History	0.85 (0.74-0.98)	1.66 (1.49-1.85)	3.18 (2.84-3.56)
MUTYH	nonHistory	0.65 (0.27-1.57)	1.25 (0.52-3)	2.37 (0.99-5.61)
	History	1.07 (0.44-2.59)	1.96 (0.81-4.67)	3.93 (1.64-9.16)
PMS2	nonHistory	0.94 (0.38-2.27)	1.89 (0.78-4.51)	3.3 (1.37-7.76)
	History	1.59 (0.65-3.84)	3.09 (1.28-7.29)	5.43 (2.28-12.49)
MSH6	nonHistory	5.35 (3.12-9.03)	9.8 (5.84-16.05)	17.7 (10.85-27.75)
	History	8.32 (4.89-13.87)	16.6 (10.12-26.22)	24.75 (15.56-37.4)
MSH2	nonHistory	7.78 (1.76-29.15)	14.95 (3.58-47.42)	NA
	History	NA	25.45 (6.61-66.02)	36.18 (10.28-79.15)
APC	nonHistory	20.45 (5.9-53.63)	34.63 (11.16-73.4)	54.2 (20.76-90.91)
	History	NA	40.87 (13.84-79.99)	NA
MLH1	nonHistory	NA	59.25 (28.03-90.24)	71.46 (37.96-98.14)
	History	49.7 (21.67-82.75)	66.19 (33.48-94.86)	90.37 (59.45-107.65)

Note:
OR = Odds ratio.; NA = represents missing data
